# Impact of osteosarcopenia on disability and mortality among Japanese older adults

**DOI:** 10.1002/jcsm.13209

**Published:** 2023-03-01

**Authors:** Hiroyuki Shimada, Takao Suzuki, Takehiko Doi, Sangyoon Lee, Sho Nakakubo, Keitaro Makino, Hidenori Arai

**Affiliations:** ^1^ Department of Preventive Gerontology, Center for Gerontology and Social Science National Center for Geriatrics and Gerontology Obu Aichi Japan; ^2^ J. F. Oberlin University Graduate Division Tokyo Japan; ^3^ National Center for Geriatrics and Gerontology Obu Aichi Japan

**Keywords:** disability, mortality, older adults, osteopenia, osteosarcopenia, sarcopenia

## Abstract

**Background:**

In clinical settings, muscle mass and bone mineral density assessments are usually performed using dual‐energy X‐ray absorptiometry (DXA), the clinical standard technique. However, DXA is often unavailable in community settings. This study aimed to determine whether osteoporosis, osteopenia (OP) and sarcopenia (SP) identified by simplified instruments are associated with the future incidence of disability and mortality and evaluate the validity of these instruments as community screening tools. We also examined osteosarcopenia (OS), defined as the coexistence of OP and SP, as a new indicator of geriatric syndromes to determine whether it has an additive effect on adverse outcome incidence compared with OP and SP alone.

**Methods:**

In total, 8995 older adults participated in the study (women: 51.7%, average age: 73.5 ± 5.4 years). Data were extracted from the Japanese national cohort study, National Center for Geriatrics and Gerontology‐Study of Geriatric Syndromes. We determined OP based on T‐scores generated based on the speed of sound, which is the time taken for ultrasound waves to go through a determined distance in the calcaneus bone. Skeletal muscle mass was evaluated using a bioimpedance analysis device. Handgrip strength and walking speed were measured as physical performance indicators. Incidences of disability and mortality were prospectively determined for 5 years.

**Results:**

The prevalence of OP, SP and OS was 45.5%, 3.9% and 7.4%, respectively. The incidence of disability in the nonOP/nonSP, OP, SP and OS groups was 6.5%, 14.9%, 20.5% and 33.5%, respectively. The incidence of mortality in the nonOP/nonSP, OP, SP and OS groups was 4.0%, 4.9%, 10.3% and 10.2%, respectively. Participants with OP (hazard ratio [HR]: 1.45, 95% confidence interval [CI]: 1.25–1.68), SP (HR: 1.38, 95% CI: 1.08–1.76) and OS (HR: 1.73, 95% CI: 1.43–2.09) had a higher risk of disability than nonOP/nonSP participants. Participants with OP (HR: 1.31, 95% CI: 1.04–1.64) and OS (HR: 1.45, 95% CI: 1.05–2.00) had a higher risk of mortality than nonOP/nonSP participants. SP was not significantly related to mortality (HR: 1.14, 95% CI: 0.90–1.45). There was no statistical interaction between OP and SP in incident disability and mortality.

**Conclusions:**

Among older adults, OS identified by bioimpedance and quantitative ultrasound assessments was associated with an increased risk of disability and mortality. Further research is needed to implement these findings in community health activities, such as setting precise cut‐off values and constructing accurate disability and mortality prediction models.

## Introduction

Osteoporosis and osteopenia (OP), along with associated fragility fractures, pose huge current and future public healthcare burden. Among the nine industrialized countries in North America, Europe, Australia and Japan, the prevalence of osteoporosis in adult Japanese females is higher than in other industrialized countries.^1^ Additionally, coupled with an aging population, the estimated number of patients with osteoporosis is significantly high.^1^

Multiple channels of communication exist between bone and muscle, including mechanical and chemical pathways.^2^ Several growth factors, interleukins and chemokines mediate the communication between the bone and muscle and vice versa, implying that the phenomenon occurring in one organ is perceived, assessed and responded to by the other.^3^ This direct mutual communication is the physiological basis for the biological link between OP and muscle wasting that constitutes the musculoskeletal syndrome.^4^ Sarcopenia (SP), the age‐related loss of muscle mass and physical performance, is increasingly becoming a medical and financial concern, such as functional disability, hospitalization and all‐cause mortality,^5^, ^6^ in aging patients and in those with OP. The pathophysiologies of OP and SP reveal overlapping features and a mechanically and biochemically (e.g., IL‐6, GH, IGF‐1, involving oestrogen, testosterone and osteocalcin) intensive and complicated interaction.^7^, ^8^ In addition, polymorphisms of the genes *glycine‐N‐acyltransferase, methyltransferase like 21C*, peroxisome proliferator‐activated receptor gamma coactivator 1‐alpha and *myocyte enhancer factor‐2* are associated with muscle atrophy and bone loss.^2^, ^8^ Mesenchymal stem cells residing in the muscle, bone and fat are implicated in OS.^2^ For instance, muscle‐derived myokines, such as myostatin, follistatin and irisin, have direct effects on bone remodelling, with the former inducing osteoclastogenesis whereas the latter two inhibit bone resorption.^8^ Due to these common associated factors of bone and muscle loss, individuals with SP and OP are classified to have osteosarcopenia (OS).^9^

This new geriatric syndrome is associated with higher incidence rates of mortality, disability, fractures and falls in older people.^10^, ^11^ These findings are based on muscle mass and bone mineral density (BMD) assessments using a dual‐energy X‐ray absorptiometry (DXA), a standard technique in clinical settings. The advantages of DXA are simple operation, fast scanning speed, low radiation dose and affordability. Despite its crucial role in clinical settings, DXA has limited accessibility for the primary screening of SP and OS in the community. Early identification of OS risk and implementation of appropriate interventions will improve the likelihood of successfully preventing adverse outcomes. Single or multi‐frequency bioimpedance analysis (BIA) has some strength of estimating muscle mass, including its low cost and the fact that it is an easy,^12^ safe and non‐invasive method.^13^ Thus, it is a useful tool that can be used in most environments^14^ and does not require highly trained personnel.^15^ These strengths are an important advantage for measuring muscle mass as a primary screening in the community.

In a simple measurement of bone status, quantitative ultrasound (QUS) classifies bone health based on changes in the speed of sound (SOS; expressed in metres per second), which is the time it takes for an ultrasound wave to pass a certain distance across the calcaneus bone, and a T‐score generated based on SOS. The association between DXA and QUS reportedly demonstrates a 90% confidence level in specificity and sensitivity, suggesting that BMD assessment by QUS predicts future fracture risk.^16^ QUS devices are ideal for the primary screening of OP in the community because these are lightweight, portable, inexpensive, have a short measurement time and lack radiation exposure. However, it is unclear whether SP and OP identified by BIA and QUS have sufficient predictive validity for the incidence of disability and death in older adults.

This study aimed to determine whether OP and SP, identified by simplified instruments, are associated with the future incidence of disability and death and evaluate the validity of these instruments as community screening tools. We also examined OS as a new indicator of geriatric syndromes and whether it has an additive effect on adverse outcome incidence compared with OP and SP alone. We hypothesized that OS is associated with the incidence of disability and death and that its impact is higher than OP and SP alone. The present study followed a 5‐year observational, prospective cohort design, using the database of the National Center for Geriatrics and Gerontology‐Study of Geriatric Syndromes (NCGG‐SGS).

## Methods

### Participants

**Figure 1:**
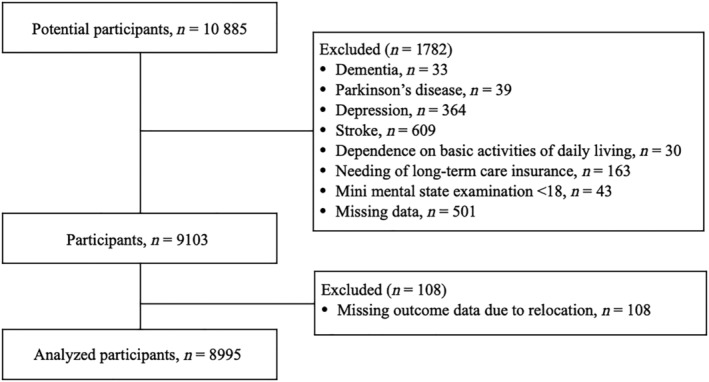
Participant flow diagram.

This nationwide study included 10 885 older adults aged ≥65 years and enrolled in NCGG‐SGS, a national cohort study in Japan. The participants were recruited from Obu and Nagoya City. The inclusion criteria were as follows: aged ≥65 years at the time of examination (2011 to 2012 in Obu and 2013 to 2014 in Nagoya) and residing in Obu or Nagoya City. Because certain conditions may present features associated with a disability,^17^ from the baseline, we excluded participants with dementia (*n* = 33), Parkinson's disease (*n* = 39), depression (*n* = 364), stroke (*n* = 609), functional decline in activities of daily living (ADLs) (*n* = 30), certified long‐term care insurance (LTCI) (*n* = 163), a Mini Mental State Examination (MMSE)^18^ score < 18 (*n* = 43) and missing data values (*n* = 501). Moreover, those who moved elsewhere (*n* = 108) during the follow‐up period were excluded due to missing outcome data. In total, 1890 patients were excluded (*Figure* *1*), and 8995 patients (mean age 73.5 ± 5.4 years, 51.7% women) were included in this study. The Ethics Committee of NCGG approved the study protocol (registration number: 1067‐3), and we obtained informed consent from all the participants prior to their inclusion.

### Assessment of osteopenia

QUS measurements were performed by a well‐trained evaluator using a gel‐based CM‐200 bone ultrasound device (Furuno, Nishinomiya, Hyogo, Japan). This device utilizes SOS (metres per second) to estimate the bone health status. This study utilized T‐scores generated based on the SOS to classify the bone health status of the participants. The T‐scores were assigned based on a reference of the Japanese sample population as provided by the manufacturer. A T‐score ≤ −2.5 represents a high risk for osteoporosis; < −1 but > −2.5 indicates OP; and > −1.0 indicates a low risk for osteoporosis.^19^ The use of WHO T‐score cut‐off values for osteoporosis and OP in QUS remains controversial because the cut‐off values of < −1 and ≤ −2.5 are established based on DXA and the skeletal properties examined are different between QUS and DXA.^20^ Therefore, to identify participants with suboptimal bone health (i.e., OP), this study used the CM‐200 optimal QUS T‐score based on sex. This method followed previous studies that identified a relationship between CM‐200 and DXA.^21^ The findings showed that in men, a cut‐off value < −1.32 improved the performance of the CM‐200 in recognizing OP compared with a cut‐off value < −1.00, and a cut‐off value < −1.61 improved the performance of the CM‐200 in recognizing osteoporosis compared with a cut‐off value ≤ −2.50. In women, a cut‐off value < −1.37 improved the performance of the CM‐200 in recognizing OP compared with a cut‐off value < −1.00, and a cut‐off value < −1.43 improved the performance of the CM‐200 in recognizing osteoporosis compared with a cut‐off value ≤ −2.50.^21^ We set the T‐score cut‐off for osteoporosis/OP at −1.61 for men and −1.43 for women. Participants with scores below these cut‐off values were considered to have a reduced BMD that indicated at least OP.

### Assessment of sarcopenia

SP was assessed based on muscle strength, muscle mass and physical performance according to the recommendations of the European Working Group on Sarcopenia in Older People (EWGSOP), which revised its operational definition of SP (EWGSOP2).^22^ However, as our participants were Japanese, we adopted the cut‐off values recommended by the Asian Working Group for Sarcopenia for the variables of interest. Specifically, low muscle strength is defined by a handgrip strength of <28 kg in men and <18 kg in women; low muscle mass is defined by a skeletal muscle mass index (SMI) of <7.0 kg/m^2^ in men and 5.7 kg/m^2^ in women; and poor physical performance is defined by a walking speed of <1.0 m/s in both men and women.^23^

Skeletal muscle mass was assessed using a multi‐frequency bioelectrical impedance analyser (MC‐980A; Tanita Corp., Tokyo, Japan), which is a tool used to assess whole and segmental body composition. The surface of the hand electrode was placed on each participant's five fingers, while a circular foot electrode was placed on each participants' heel and front foot. During measurements, the participants were asked to hold out their arms and legs to prevent any other body part from touching the electrodes. The bioelectrical impedance analyser used six electrical frequencies: 1, 5, 50, 250, 500 and 1000 kHz. Based on segmental body composition measurements, appendicular skeletal muscle mass was calculated using the following formula developed by NCGG: appendicular skeletal muscle mass (men) = (0.197 × height^2^/50 kHz resistance) + (0.179 × weight) − 0.019 and appendicular skeletal muscle mass (women) = (0.221 × height^2^/50 kHz resistance) + (0.117 × weight) + 0.881.^24^

The skeletal muscle mass measurements were converted to SMIs by dividing the participants' muscle masses by their squared heights (kilograms per square metre). Handgrip strength was assessed using a Smedley‐type handheld dynamometer (Takei Scientific Instruments Co., Ltd., Niigata, Japan). Grip strength was measured in a standing position with the elbow extended. The participants held the handheld dynamometer with the PIP joint of the index finger at 90°. The highest value of two assessments using the dominant hand was used for analysis. Walking speed was measured by asking the participants to walk on a flat, straight surface at a comfortable pace. Two markers were used to indicate the start and the end of a 2.4‐m walking path. To ensure that the participants will walk at a comfortable pace upon reaching the timed path, the participants were asked to walk through an additional 2‐m section before the start marker. To ensure a consistent walking pace throughout the timed path, the participants were asked to continue walking through an additional 2‐m section after the end of the timed path. Walking time was measured in seconds using a stopwatch, and the participants' walking speeds (metres per second) were calculated.

EWGSOP2 considers low muscle strength a key characteristic of SP, diagnoses low muscle quantity and quality as SP and identifies poor physical performance as an indicator of severe SP. EWGSOP2 recommends a muscle strength test, such as measuring handgrip strength, to initially detect SP, identifying individuals with probable SP. Low muscle mass or quality confirms SP diagnosis. However, low muscle strength alone is only enough to start interventions^22^ because the adverse effects of untreated muscle weakness, even without low muscle mass and quality, can greatly influence healthcare costs.^25^ Assessing probable SP is crucial for SP prevention and improvement of health.^26^ Therefore, in this study, SP included probable SP with decreased grip strength.

### Incident of functional disability and mortality

In this study, the participants were tracked monthly to clarify new public LTCI certifications, which are recorded and managed by the Japanese LTCI system per municipality. LTCI certification details have been reported by a previous study.^27^ In Japan, all individuals aged ≥65 years, as well as those aged 40–64 years with age‐related diseases, are immediately eligible for LTCI benefits. When an individual applies for LTCI benefits in a municipality, an authorized care manager examines the applicant's physical and mental status through a standardized questionnaire. A certification committee comprising medical doctors and nurses then determines the required level of care. These estimates are based on the required duration of anticipated care and input from the applicant's family physician. Certification category ‘Support Level 1 or 2’ indicates a need for ADL support, whereas category ‘Care Level 1 through 5’ indicates a need for continuous care.^27^ In this study, disability was defined as an LTCI certification of any level. We defined disability onset as the time at which a participant was issued an LTCI certificate for the requirement of support or care and followed up to assess disability incidence for 60 months.

We also followed up the incidence of all‐cause mortality for 1800 days, using residence records and local government data. We collected monthly data from the municipal government and identified the incidence of death.

### Potential confounding factors

We selected age, sex, hypertension, heart disease, pulmonary disease, diabetes, osteoarthritis of the knee, body mass index (BMI), walking speed, physical activity, MMSE and the 15‐item version of the Geriatric Depression Scale (GDS‐15)^28^ as possible confounding factors associated with ADL decline (*Table* *1*). Medical information was obtained via interview‐based surveys. We evaluated physical inactivity using the following questions about time spent for sports and exercise: (i) ‘Do you engage in moderate levels of physical exercise or sports aimed at health?’ and (ii) ‘Do you engage in low levels of physical exercise aimed at health?’ The participants who answered ‘no’ to both of these questions were classified as physically inactive.^29^

**Table 1:** Demographic characteristics of the study participants stratified by osteosarcopenia status

	nonOsteopenia/nonSarcopenia (*n* = 3887)	Osteopenia (*n* = 4091)	Sarcopenia (*n* = 348)	Osteosarcopenia (*n* = 669)	*P* value
Age, years	72.0 ± 4.9	73.8 ± 5.3	77.1 ± 5.1	78.3 ± 5.3	<0.01
Sex, female (*n*, %)	1192, 30.7	2923, 71.4	92, 26.4	441, 65.9	<0.01
Hypertension, yes (*n*, %)	1746, 44.9	1837, 44.9	161, 46.3	317, 47.4	0.64
Heart disease, yes (*n*, %)	607, 15.6	660, 16.1	79, 22.7	126, 18.8	<0.01
Pulmonary disease, yes (*n*, %)	487, 12.5	601, 14.7	58, 16.7	118, 17.6	<0.01
Diabetes, yes (*n*, %)	563, 14.5	401, 9.8	81, 23.3	99, 14.8	<0.01
Osteoarthritis, yes (*n*, %)	517, 13.3	800, 19.6	64, 18.4	191, 28.6	<0.01
Body mass index	23.7 ± 2.9	22.8 ± 3.2	23.0 ± 3.0	22.1 ± 3.0	<0.01
Walking speed, m/s	1.2 ± 0.2	1.1 ± 0.2	1.0 ± 0.2	1.0 ± 0.2	<0.01
Physical inactivity, yes (*n*, %)	958, 24.6	992, 24.2	79, 22.7	176, 26.3	0.58
MMSE, point	26.3 ± 2.5	26.3 ± 2.5	25.5 ± 2.7	25.4 ± 2.7	<0.01
GDS, point	2.5 ± 2.5	2.8 ± 2.5	3.4 ± 3.0	3.5 ± 2.8	<0.01
SOS, T‐score	−0.9 ± 0.6	−2.0 ± 0.3	−1.0 ± 0.5	−2.1 ± 0.4	<0.01
Grip strength, kg	30.7 ± 7.4	24.9 ± 6.7	22.2 ± 4.7	18.5 ± 4.8	<0.01
Skeletal muscle index, kg/m^2^	7.4 ± 1.1	6.6 ± 0.9	7.2 ± 1.0	6.4 ± 0.9	<0.01

*Note*: Data are presented as mean ± standard deviation for continuous variables or number and percentage for categorical variables.

Abbreviations: GDS, Geriatric Depression Scale; MMSE, Mini Mental State Examination; SOS, speed of sound.

### Statistical analysis

Baseline clinical and demographic characteristics were compared according to OS and disability or mortality status through one‐way analysis of variance, t‐tests and chi‐squared tests. Participants who died during the study period were excluded from the univariate analyses of disability incidence. We calculated the cumulative incidence of disability and mortality for the follow‐up period. The participants were divided into the nonOP/nonSP, OP, SP and OS, which included both OP and SP, groups. We used adjusted, standardized residuals to identify the effect of OS on disability and mortality incidences through chi‐squared tests. The adjusted, standardized residuals followed the t distribution, with *P* > 1.96, *P* < 0.05 and *P* > 2.56, and *P* < 0.01. Cox proportional hazards regression models were adjusted for potential confounding factors and used to determine the association between OS status and the incidence of disability and mortality. We estimated adjusted hazard ratios (HRs) and the corresponding 95% confidence intervals (CIs) for the disability incidence. To rule out reverse causality (i.e., functional decline or mortality causing OS), lagged analyses were conducted to exclude participants who developed incident disability and mortality within 12 months. To test for interactions in the relationships of OP and SP with disability and mortality, multivariable binary logistic regression analyses were performed to examine the associations of continuous (handgrip strength, SMI and T‐score of SOS) and categorical (OP and SP) OS components with the likelihood of disability and mortality during the follow‐up period. These analyses were adjusted for variables, including potential confounding factors, and the continuous variable analysis was further adjusted for all other components of OS. The interaction terms included in these models were as follows: for the categorical variable model, OP × SP; for the continuous variable model, T‐score of SOS × handgrip strength, T‐score of SOS × SMI. Data management and statistical analyses were performed using the IBM SPSS Statistics Version 24.0 software package (IBM, Tokyo, Japan). The significance level was set at *P* < 0.05 for all analyses.

## Results

**Figure 2:**
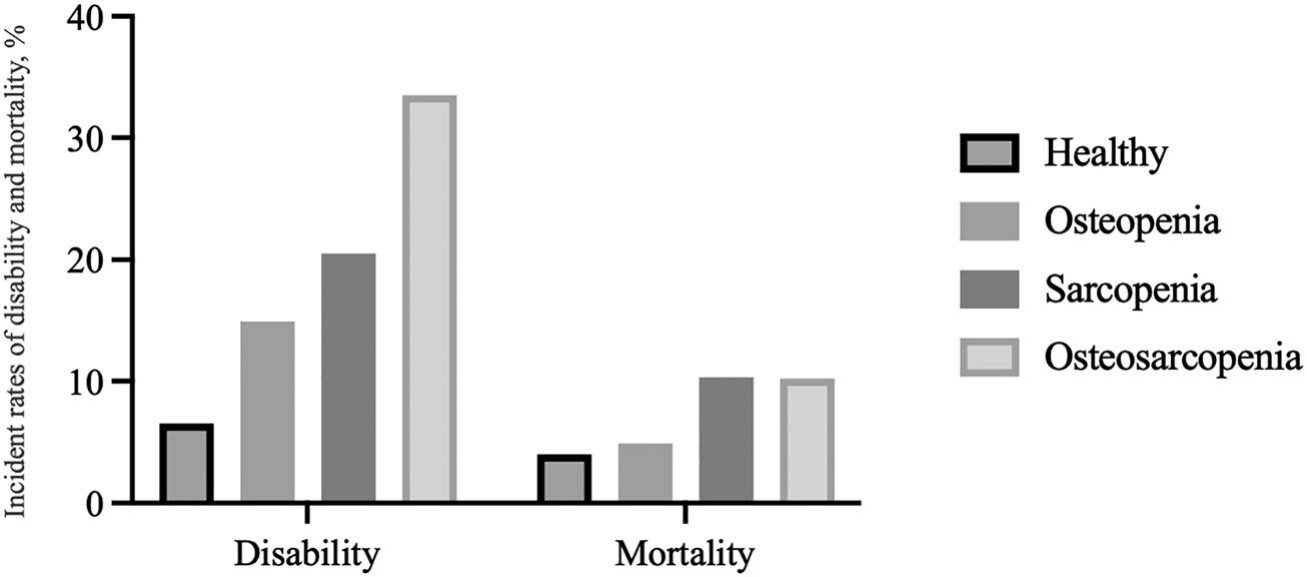
Incidence rates of disability and mortality according to osteosarcopenia status.

Among the older adults registered in NCGG‐SGS, 4091 (45.5%) had OP, 348 (3.9%) had SP and 669 (7.4%) had OS. Further, 1338 (14.9%) had a certified disability and 463 (5.1%) died during the 5‐year follow‐up period. The disability and mortality rates were 32.2 (95% CI: 30.6–34.0) and 10.5 (95% CI: 9.6–11.5) per 1000 person‐years, respectively. The incidence of disability in the nonOP/nonSP, OP, SP and OS groups was 6.5%, 14.9%, 20.5% and 33.5%, respectively (*Figure* *2*). The incidence of mortality in the nonOP/nonSP, OP, SP and OS groups was 4.0%, 4.9%, 10.3% and 10.2%, respectively (*Figure* *2*). Residual analyses revealed that the OP (*P* < 0.01), SP (*P* < 0.01) and OS (*P* < 0.01) groups had significantly more participants with a certified disability. Additionally, the SP (*P* < 0.01) and OS (*P* < 0.01) groups had significantly more participants who died (*Figure* *2*).

*Table* *1* presents the means and frequencies of factors associated with disability and mortality incidence (potential confounders) among participants stratified by the OS status. Significant differences were observed between the SP subgroups in terms of average age and sex; presence of heart disease, pulmonary disease, diabetes and osteoarthritis; BMI and walking speed measurements; and MMSE and GDS scores. Significant differences were also noted in SOS T‐score, grip strength and SMI, which are components of OS (*Table* *1*).

Additionally, significant differences were found between the disability status groups in all variables except for BMI (Supporting Information, *Appendix* *S1*). Significant differences were also observed between the mortality status groups in terms of average age and sex; presence of heart disease, pulmonary disease and diabetes; BMI and walking speed measurements; physical inactivity; and MMSE, GDS and SOS T‐scores (Supporting Information, *Appendix* *S2*).

**Figure 3:**
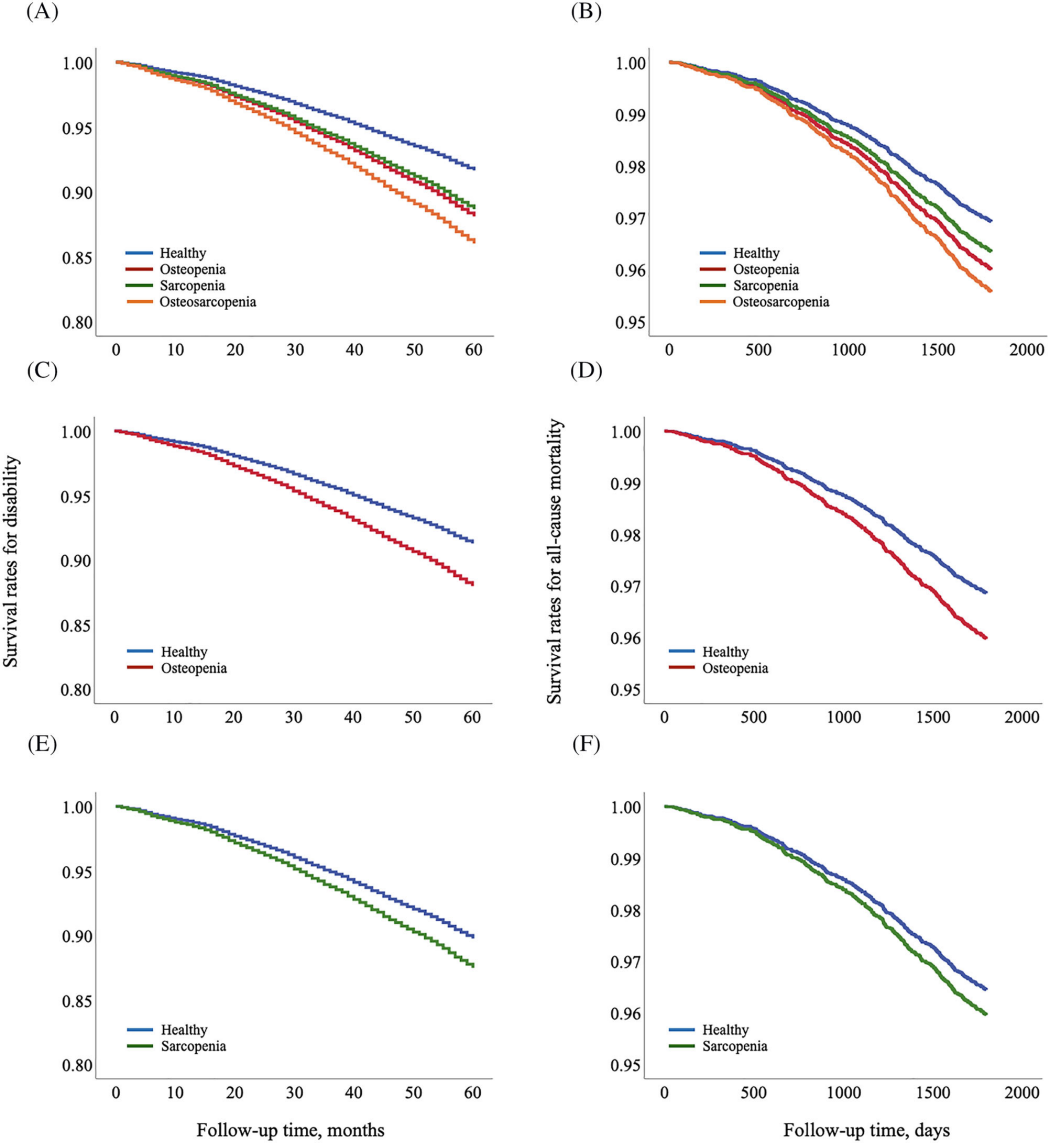
Cox survival estimates for disability and mortality incidences according to osteosarcopenia status. (A) Hazard estimates of disability occurrence in the osteopenia, sarcopenia and osteosarcopenia groups compared with that of the nonOP/nonSP group. (B) Hazard estimates of mortality occurrence in the osteopenia, sarcopenia and osteosarcopenia groups compared with that of the nonOP/nonSP group. (C) Hazard estimates of disability incidence in the osteopenia group compared with that of the nonOP/nonSP group. (D) Hazard estimates of mortality incidence in the osteopenia group compared with that of the nonOP/nonSP group. (E) Hazard estimates of disability incidence in the sarcopenia group compared with that of the nonOP/nonSP group. (F) Hazard estimates of mortality incidence in the sarcopenia group compared with that of the nonOP/nonSP group.

The Cox proportional hazards regression models revealed associations between OP (HR: 1.45, 95% CI: 1.25–1.68), SP (HR: 1.38, 95% CI: 1.08–1.76) and OS (HR: 1.73, 95% CI: 1.43–2.09) and disability incidence (*Table* *2* and *Figure* *3* *A*). Among the potential confounding factors, older age, female sex, diabetes, low walking speed and low MMSE and GDS scores were positively correlated with the incidence of disability (*Table* *2*). The models also revealed associations between OP (HR: 1.31, 95% CI: 1.04–1.64) and OS (HR: 1.45, 95% CI: 1.05–2.00) and mortality incidence. However, no significant association was found between SP and mortality incidence (HR: 1.19, 95% CI: 0.82–1.74) (*Table* *2* and *Figure* *3* *B*).

**Table 2:** Hazard ratios for disability and mortality according to osteosarcopenia status

	Disability		Mortality		Disability with 12‐month lag		Mortality with 12‐month lag	
	Hazard ratio (95% CI)	*P* value	Hazard ratio (95% CI)	*P* value	Hazard ratio (95% CI)	*P* value	Hazard ratio (95% CI)	*P* value
Age, years	1.11 (1.10–1.12)	<0.01	1.09 (1.07–1.11)	<0.01	1.11 (1.1–1.12)	<0.01	1.09 (1.07–1.11)	<0.01
Sex, male	0.84 (0.75–0.95)	<0.01	2.51 (2.02–3.12)	<0.01	0.86 (0.75–0.98)	0.02	2.47 (1.97–3.09)	<0.01
Hypertension, yes	1.11 (1.00–1.25)	0.06	0.90 (0.74–1.08)	0.25	1.13 (1.01–1.28)	0.04	0.88 (0.73–1.08)	0.22
Heart disease, yes	1.12 (0.98–1.28)	0.09	1.05 (0.83–1.32)	0.69	1.10 (0.95–1.27)	0.20	1.03 (0.81–1.30)	0.83
Pulmonary disease, yes	1.12 (0.97–1.29)	0.13	1.30 (1.04–1.62)	0.02	1.15 (0.99–1.34)	0.07	1.28 (1.01–1.62)	0.04
Diabetes, yes	1.20 (1.03–1.39)	0.02	1.18 (0.92–1.52)	0.19	1.21 (1.03–1.41)	0.02	1.21 (0.93–1.57)	0.15
Osteoarthritis, yes	1.09 (0.95–1.24)	0.22	0.82 (0.63–1.07)	0.15	1.09 (0.95–1.26)	0.24	0.83 (0.63–1.10)	0.20
Body mass index	1.00 (0.98–1.02)	0.84	0.94 (0.91–0.97)	<0.01	1.00 (0.98–1.02)	0.92	0.94 (0.91–0.98)	<0.01
Walking speed, m/s	0.16 (0.12–0.21)	<0.01	0.41 (0.25–0.66)	<0.01	0.17 (0.13–0.24)	<0.01	0.40 (0.24–0.66)	<0.01
Physical inactivity, yes	1.07 (0.95–1.20)	0.29	0.99 (0.81–1.22)	0.94	1.09 (0.96–1.25)	0.18	1.01 (0.81–1.25)	0.96
MMSE, point	0.95 (0.94–0.97)	<0.01	0.96 (0.93–1.00)	0.03	0.96 (0.94–0.98)	<0.01	0.97 (0.93–1.00)	0.07
GDS, point	1.05 (1.03–1.07)	<0.01	1.06 (1.03–1.09)	<0.01	1.06 (1.04–1.08)	<0.01	1.06 (1.03–1.10)	<0.01
**Osteosarcopenia status**								
nonOsteopenia/nonSarcopenia	1	<0.01*	1	0.07*	1	<0.01*	1	0.15*
Osteopenia	1.45 (1.25–1.68)	<0.01	1.31 (1.04–1.64)	0.02	1.44 (1.24–1.68)	<0.01	1.27 (1.00–1.61)	0.05
Sarcopenia	1.38 (1.08–1.76)	<0.01	1.19 (0.82–1.74)	0.36	1.29 (0.99–1.68)	0.06	1.08 (0.72–1.61)	0.72
Osteosarcopenia	1.73 (1.43–2.09)	<0.01	1.45 (1.05–2.00)	0.02	1.65 (1.34–2.02)	<0.01	1.38 (0.99–1.93)	0.06

Abbreviations: CI, confidence interval; GDS, Geriatric Depression Scale; MMSE, Mini Mental State Examination.

* *P* for trend.

The models were adjusted for confounders by classifying the participants into the nonOP/nonSP and OP groups, revealing significant associations between OP and disability (HR: 1.40, 95% CI: 1.23–1.60) and mortality (HR: 1.29, 95% CI: 1.05–1.58) incidences (*Figure* *3* *C,D*). Likewise, classifying the participants into the nonOP/nonSP and SP groups revealed significant associations between SP and disability incidence (HR: 1.24, 95% CI: 1.08–1.41). However, no significant relationship was found between SP and mortality incidence (HR: 1.14, 95% CI: 0.90–1.45) (*Figure* *3* *E,F*).

In the 12‐month lagged analyses, the associations between incident disability and OP (HR: 1.44, 95% CI: 1.24–1.68) and OS (HR: 1.65, 95% CI: 1.34–2.02) remained significant. However, the association between incident disability and SP did not reach significance (HR: 1.29, 95% CI: 0.99–1.68) (*Table* *2*). Additionally, the association between mortality and OP (HR: 1.27, 95% CI: 1.00–1.61) remained significant. However, the significance of OS marginally failed to reach significance in lag analysis (HR: 1.38, 95% CI: 0.99–1.93).

*Table* *3* presents the odds ratios (ORs) for disability and mortality according to components of OS at baseline. After adjustment for potential confounders, the T‐score of SOS and the grip strength were significantly associated with reduced likelihood of disability. Only higher T‐score of SOS predicted lower likelihood of mortality. There were no significant interactions between the T‐score of SOS and components of SP for disability and mortality. In OP and SP categories, only OP was significantly associated with increased likelihood of disability and mortality. The interaction between OP and SP approached no significance for likelihood of disability and mortality.

**Table 3:** Associations of osteosarcopenia and its components with the disability and mortality rates

	Disability		Mortality	
Osteosarcopenia components	Odds ratio (95% CI)	Interaction term with T‐score of SOS (*P* value)	Odds ratio (95% CI)	Interaction term with T‐score of SOS (*P* value)
**T‐score of SOS**	**0.74 (0.67–0.83)**	—	**0.80 (0.68–0.93)**	—
SMI	1.28 (1.00–1.66)	0.44	0.94 (0.65–1.37)	0.66
**Grip strength**	**0.95 (0.93–0.98)**	0.59	0.97 (0.93–1.00)	0.38

Osteosarcopenia categories	Odds ratio (95% CI)	Interaction term (*P* value)	Odds ratio (95% CI)	Interaction term (*P* value)
**Osteopenia**	**1.47 (1.24–1.73)**	0.80	**1.34 (1.05–1.70)**	0.62
Sarcopenia	1.32 (0.98–1.79)		1.24 (0.83–1.86)	

*Note*: Bold values are significant.

Abbreviations: CI, confidence interval; SMI, skeletal muscle mass index; SOS, speed of sound.

## Discussion

This study presented original data on OS from 8995 older adult community‐dwellers. The results of this study suggested that disability is associated with OP, SP and OS. However, mortality may not be associated with SP. Further, muscle weakness was unlikely to predict future mortality risk, and low BMD or the coexistence of low BMD and muscle weakness was closely associated with mortality in older adults.

SP develops due to multiple factors, such as nutritional status, inflammatory processes related to aging, availability of intramuscular fat, genetic predisposition and reduced physical activity.^30^ Previous studies have determined the association between SP and functional decline and disability.^31^, ^32^ Participants were classified based on SP status in the Cardiovascular Health Study as follows: normal SMI (men: ≥10.76 kg/m^2^, women: ≥6.76 kg/m^2^), moderate SP (men: 8.51–10.75 kg/m^2^, women: 5.76–6.75 kg/m^2^) and severe SP (men: ≤8.50 kg/m^2^, women: ≤5.75 kg/m^2^). During the 8‐year follow‐up period, participants with severe SP had a 27% higher risk of developing disability; no statistical difference in disability risk was observed between participants with moderate SP and those with normal muscle mass.^32^ Similarly, this study showed an increased risk of developing a disability, even in participants with probable SP.

Recently, a systematic review revealed that SP significantly increased the risk of mortality, independent of population and SP definition (HR: 2.00, 95% CI: 1.71–2.34).^33^ Further, a meta‐analysis reported that SP was associated with an increased risk of all‐cause mortality (OR: 3.64, 95% CI: 2.94–4.51) and functional decline (OR: 2.58, 95% CI: 1.33–4.99).^34^ Although these findings are based on the EWGSOP criteria, the study results are not presented according to SP severity. Moreover, based on the recommendations of the new EWGSOP2 consensus, older adults with muscle mass loss and gait speed reduction but unchanged strength are classified as normal. As such, SP is diagnosed only through muscular strength evaluation and ruled out by preserved grip strength, even without the evaluation of the other components. Thus, it is worth questioning whether the current recommendations for SP diagnosis are adequate to classify mortality risk in older adults, especially because the diagnostic criteria in each study may change the pattern of associations.^35^ Using EWGSOP2, Bachettini et al. reported that older adults with severe SP have an increased risk of mortality compared with those without SP (HR: 4.11, 95% CI: 1.88–9.00). However, no statistically significant association was found between probable and confirmed SP and mortality risk.^36^ Likewise, our study results showed that low muscle strength was not independently associated with mortality.

The Cox proportional hazards regression models revealed that older adults with OP were associated with a significantly higher risk of disability and mortality than the older adults without OP and SP. Osteoporotic fractures are a rising global public health concern. Its projected incidence increases with the aging of the population.^37^ The burden of fractures relates to cost, disability and associated mortality.^38^ Specifically, premature mortality following hip and vertebral fractures is gaining recognition.^38^ However, the mechanism behind the fracture–mortality association remains unclear. Some studies suggest that it is largely related to underlying health status or comorbidities,^39^ whereas others have found little to no evidence supporting this.^40^ In our study, OP was associated with disability and mortality even after adjustment for several health issues and chronic diseases, and this association remained significant in the lagged sensitivity analysis.

In previous population studies, QUS indices are significantly associated with bone mass, bone microarchitecture and fracture risk.^41^ According to the International Society for Clinical Densitometry, the calcaneus is the only anatomical position for QUS measurement recommended for osteoporosis screening,^20^ because of the primarily cancellous bone composition, with little soft tissue and a large parallel surface.^42^ It is also radiation‐free, portable and simple to operate, making it suitable for extensive clinical use. QUS, which is easily accessible, allows for the early screening of OP in the community and can be used to examine strategies to reduce the incidence of disability and death among high‐risk older adults.

OS has become increasingly relevant in recent years because it is associated with adverse outcomes such as falls, fractures, frailty, functional disability and mortality risk.^2^, ^10^, ^11^ Bone and muscle are closely related, and multiple signalling pathways, including chemical and mechanical pathways, ensure communication between these two organs.^2^ The bone and muscle interact with each other through numerous growth factors, interleukins and chemokines, indicating that a phenomenon occurring in one organ is perceived, assessed and responded to by the other.^3^ This direct intercommunication supports a biological association between OP and SP, accounting for a geriatric syndrome rather than a simple epidemiological association. In our study, older adults with OS had significantly higher HRs for disability and mortality incidences compared with the older adults without OP and SP and higher HRs compared with those with OP or SP alone. Thus, healthcare providers are advised to perform both muscle and bone assessments to evaluate disability and mortality risks and provide suitable interventions to high‐risk individuals, particularly older adults with OS. However, the relationship between mortality and OS was not significant in the lag analysis; whether OS is a causal factor in mortality needs further exploration. Finally, the QUS measurements were made at a single location and at a single time point to estimate the bone status, but multiple locations and multiple time points could have improved the accuracy of the OP determination.

There are several further limitations in generalizing the study findings. First, NCGG‐SGS participants were not randomly selected, which may have underestimated the SP prevalence. The cohort participants were generally healthy older adults with access to health screenings from their home. Second, it was not possible to contact family members or other informants to verify participants' medical history or ADLs. Third, other potential confounders, such as drug therapy and diet quality, could not be assessed. Fourth, medical diagnoses were obtained from self‐reports and not medical records. Finally, the QUS measurements were made at a single location and at a single time point to estimate bone status, but multiple locations and multiple time points could have improved the accuracy of the OP determination. Despite the limitations, the strengths of this study were its prospective cohort design, which can address the causality between OS and disability and mortality, large community cohort and comprehensive geriatric assessment of OS.

## Conclusions

This study revealed that OS defined by BIA and QUS was associated with an increased risk of disability and mortality among older adults. Further research is needed to implement these findings in community health activities, such as setting better cut‐off values and constructing accurate disability and mortality prediction models through machine learning.

## Funding

This work received financial support via the Health Labour Sciences Research Grant from the Japanese Ministry of Health, Labour and Welfare (H23‐tyoujyu‐ippan‐001, H24‐tyoujyu‐ippan‐004), Research Funding for Longevity Sciences from the National Center for Geriatrics and Gerontology (22‐16, 24‐18, 25‐26, 26‐33, 27‐22), Obu City Local Government, Grant‐in‐Aid for Scientific Research (B) from the Japan Society for the Promotion of Science (23300205) and Strategic Basic Research Programs (RISTEX Redesigning Communities for Aged Society) from the Japan Science and Technology Agency. The funding sources played no role in the design or conduct of the study; collection, management, analysis or interpretation of the data; or preparation, review or approval of the manuscript.

## Conflict of interest

The authors declare no conflict of interest.

## Supporting information


**Appendix S1.** **Supplement 1.** Clinical and demographic characteristics of the study participants stratified by disability incidence

**Appendix S2.** **Supplement 2.** Clinical and demographic characteristics of the study participants stratified by mortality
