# Supplementary material for: Impact of osteosarcopenia on disability and mortality among Japanese older adults
Source: J Cachexia Sarcopenia Muscle. 2023 Mar 1;14(2):1107–16. doi: 10.1002/jcsm.13209 (PMC10067490; doi:10.1002/jcsm.13209)
Supplement: Supplementary file 1 — Appendix S1. Supplement 1. Clinical and demographic characteristics of the study participants stratified by disability incidence [file JCSM-14-1107-s001.pdf]

Title: Impact of osteosarcopenia on disability and mortality among Japanese older adults

Journal: Journal of Cachexia, Sarcopenia and Muscle

Hiroyuki Shimada[1], Takao Suzuki[1,2], Takehiko Doi[1], Sangyoon Lee[1], Sho Nakakubo[1], Keitaro Makino[1], Hidenori Arai[3]

[1]Department of Preventive Gerontology, Center for Gerontology and Social Science, National Center for Geriatrics and Gerontology, Aichi, Japan

[2] J. F. Oberlin University Graduate Division, Tokyo, Japan

[3]National Center for Geriatrics and Gerontology, Aichi, Japan

*Corresponding author:

Hiroyuki Shimada

Center for Gerontology and Social Science, National Center for Geriatrics and Gerontology,

7-430, Morioka-cho, Obu City, Aichi Prefecture, 474-8511, Japan

Tel: +81-562-44-5651

Fax: +81-562-46-8294

E-mail: shimada@ncgg.go.jp

Supplement 1. Clinical and demographic characteristics of the study participants stratified by disability incidence

	Participants without disability (n = 7,441)	Participants with disability (n = 1,087)	P value
Age, years	72.6 ± 4.9	78.0 ± 5.6	＜0.01
Sex, female	3818, 51.3	685, 63.0	＜0.01
Hypertension, yes	3258, 43.8	593, 54.6	＜0.01
Heart disease, yes	1148, 15.4	227, 20.9	＜0.01
Pulmonary disease, yes	984, 13.2	181, 16.7	＜0.01
Diabetes, yes	900, 12.1	168, 15.5	＜0.01
Osteoarthritis, yes	1209, 16.2	296, 27.2	＜0.01
Body mass index	23.2 ± 3.0	23.2 ± 3.4	0.89
Walking speed, n	1.2 ± 0.2	1.0 ± 0.2	＜0.01
Physical inactivity, n	1749, 23.5	316, 29.1	＜0.01
MMSE, point	26.4 ± 2.4	25.4 ± 2.8	＜0.01
GDS, point	2.6 ± 2.5	3.5 ± 2.8	＜0.01
SOS T-score	-1.4 ± 0.7	-1.8 ± 0.7	＜0.01
Grip strength, kg	27.4 ± 7.8	23.1 ± 6.9	＜0.01

Skeletal muscle index	7.1 ± 1.0	7.0 ± 0.9	＜0.01
