# Supplementary material for: Impact of osteosarcopenia on disability and mortality among Japanese older adults
Source: J Cachexia Sarcopenia Muscle. 2023 Mar 1;14(2):1107–16. doi: 10.1002/jcsm.13209 (PMC10067490; doi:10.1002/jcsm.13209)
Supplement: Supplementary file 2 — Appendix S2. Supplement 2. Clinical and demographic characteristics of the study participants stratified by mortality [file JCSM-14-1107-s002.pdf]

Title: Impact of osteosarcopenia on disability and mortality among Japanese older adults

Journal: Journal of Cachexia, Sarcopenia and Muscle

Hiroyuki Shimada[1], Takao Suzuki[1,2], Takehiko Doi[1], Sangyoon Lee[1], Sho Nakakubo[1], Keitaro Makino[1], Hidenori Arai[3]

[1]Department of Preventive Gerontology, Center for Gerontology and Social Science, National Center for Geriatrics and Gerontology, Aichi, Japan

[2] J. F. Oberlin University Graduate Division, Tokyo, Japan

[3]National Center for Geriatrics and Gerontology, Aichi, Japan

*Corresponding author:

Hiroyuki Shimada

Center for Gerontology and Social Science, National Center for Geriatrics and Gerontology,

7-430, Morioka-cho, Obu City, Aichi Prefecture, 474-8511, Japan

Tel: +81-562-44-5651

Fax: +81-562-46-8294

E-mail: shimada@ncgg.go.jp

Supplement 2. Clinical and demographic characteristics of the study participants stratified by mortality

	Participants without mortality (n = 8,532)	Participants with mortality (n = 463)	P value
Age, years	73.3 ± 5.3	77.3 ± 6.0	<0.01
Sex, female	4503, 52.8	145, 31.3	＜0.01
Hypertension, yes	3852, 45.1	209, 45.1	1.00
Heart disease, yes	1375, 16.1	97, 21.0	＜0.01
Pulmonary disease, yes	1166, 13.7	98, 21.2	＜0.01
Diabetes, yes	1068, 12.5	76, 16.4	0.01
Osteoarthritis, yes	1505, 17.6	67, 14.5	0.08
Body mass index	23.2 ± 3.1	22.5 ± 3.4	<0.01
Walking speed, n	1.2 ± 0.2	1.0 ± 0.2	<0.01
Physical inactivity, n	2066, 24.2	139, 30.0	＜0.01
MMSE, point	26.2 ± 2.5	25.3 ± 2.8	<0.01
GDS, point	2.7 ± 2.5	3.6 ± 2.9	<0.01
SOS T-score	-1.5 ± 0.7	-1.6 ± 0.7	<0.01
Grip strength, kg	26.8 ± 7.8	26.2 ± 7.5	0.10
Skeletal muscle index	7.1 ± 1.0	7.1 ± 1.0	0.17
